# PFKFB3 Inhibition Attenuates Oxaliplatin-Induced Autophagy and Enhances Its Cytotoxicity in Colon Cancer Cells

**DOI:** 10.3390/ijms20215415

**Published:** 2019-10-30

**Authors:** Siyuan Yan, Nan Zhou, Deru Zhang, Kaile Zhang, Wenao Zheng, Yonghua Bao, Wancai Yang

**Affiliations:** 1 Key Laboratory of Precision Oncology of Shandong Higher Education, Institute of Precision Medicine, Jining Medical University, Jining 272067, China; zhounanzn720@163.com (N.Z.); zhangdr2613@163.com (D.Z.); zhangkaile1315@163.com (K.Z.); zwa2019med@163.com (W.Z.); baoyonghua2005@126.com (Y.B.); 2 Department of Pathology, University of Illinois at Chicago, Chicago, IL 60612, USA

**Keywords:** PFKFB3, Oxaliplatin, autophagy, colorectal cancer, proliferation

## Abstract

6-Phosphofructo-2-kinase/fructose-2,6-bisphosphatase isoform 3 (PFKFB3), a glycolytic enzyme highly expressed in cancer cells, has been reported to participate in regulating metabolism, angiogenesis, and autophagy. Although anti-cancer drug oxaliplatin (Oxa) effectively inhibits cell proliferation and induces apoptosis, the growing resistance and side-effects make it urgent to improve the therapeutic strategy of Oxa. Although Oxa induces the autophagy process, the role of PFKFB3 in this process remains unknown. In addition, whether PFKFB3 affects the cytotoxicity of Oxa has not been investigated. Here, we show that Oxa-inhibited cell proliferation and migration concomitant with the induction of apoptosis and autophagy in SW480 cells. Both inhibition of autophagy by small molecule inhibitors and siRNA modification decreased the cell viability loss and apoptosis induced by Oxa. Utilizing quantitative PCR and immunoblotting, we observed that Oxa increased PFKFB3 expression in a time- and dose-dependent manner. Meanwhile, suppression of PFKFB3 attenuated both the basal and Oxa-induced autophagy, by monitoring the autophagic flux and phosphorylated-Ulk1, which play essential roles in autophagy initiation. Moreover, PFKFB3 inhibition further inhibited the cell proliferation/migration, and cell viability decreased by Oxa. Collectively, the presented data demonstrated that PFKFB3 inhibition attenuated Oxa-induced autophagy and enhanced its cytotoxicity in colorectal cancer cells.

## 1. Introduction

Colorectal cancer (CRC) is one of the major human malignant tumors, which ranks as the third most common and fourth most common cancer according to morbidity and mortality, respectively. Approximately 1.2 million new cases and 0.6 million patients directly or indirectly die of CRC per year globally [1,2]. During the past decades, the prognosis of CRC has steadily improved with the improvement of medical expertise, and the 5-year relative survival rate is nearly 65% in developed countries [3]. However, CRC tends to develop distant organ metastasis, which is one of the most major important prognostic and lethal factors [4,5]. Moreover, plenty of CRC patients are not sensitive to chemotherapy, and usually suffer from multidrug resistance (MDR), making it intractable and urgent to be solved in CRC therapy [6,7].

Accumulating evidence indicates that autophagy plays a protective effect during chemotherapy, and may be involved in drug resistance [8,9]. As a highly conversed process in maintaining homeostasis during stresses, autophagy plays double-edged sword role in tumorigenesis and cancer therapy, during which autophagy is an attempt to resist the factors that changing the cell state [10]. However, excessive autophagy may also lead to cell death, and this so-called “autophagic cell death” is considered as type II programmed cell death (PCD) [11,12]. Thus, there is no wonder that autophagy may give rise to the survival of MDR cancers under some certain conditions, but lead to cancer cell death in other cases [13]. Autophagy is induced by various stimulators, such as hypoxia, nutrient deprivation, and some kind of chemotherapeutic agents. Among the variety signaling pathways participated in regulating autophagy, the Akt-mTOR (protein kinase B-mechanistic target of rapamycin) pathway and AMPK (AMP-activated protein kinase) pathway are two essential regulators of autophagy. Generally, Akt-mTOR negatively regulates autophagy by attenuating the activity of Ulk1 (unc-51-like kinase 1), which plays an essential role in autophagy initiation [14]; whereas AMPK can activate autophagy indirectly by deactivating the Akt-mTOR pathway [15,16], or directly by inducing Ulk1 activity [17]. The rapid proliferation of cancer cells results in the lack of normal and mature vessels in the internal solid tumor, and the consequent hypoxia and nutrition deficiency microenvironment may activate the AMPK pathway and autophagy process to maintain cancer cell survival [18,19].

The alteration of metabolism in cancer cells sustains its continuous cell growth and proliferation in the stress microenvironment [20]. Even when not under a hypoxia condition, cancer cells are prone to utilize glycolysis to metabolize glucose instead of oxidative phosphorylation. This phenomenon, known as the Warburg effect, is considered to provide anabolic substrates to meet the massive demand for rapid cell proliferation [21]. Due to its lowest catalytic efficiency to generate fructose-1,6-bisphosphate (F1,6BP), the catalytic activity of 6-phosphofructo-1-kinase (PFK-1) determines the rate of glycolysis [20]. PFK-1 could be effectively allostericly activated by fructose-2,6-bisphosphate (F2,6BP), which is controlled by a family of bifunctional enzymes known as 6-phosphofructo-2-kinase /fructose-2,6-bisphosphatase (PFK-2/FBPase) [22]. Besides displaying the highest ratio of kinase/phosphatase, PFKFB3 is highly expressed in a variety of cancer cells, and can be induced by hypoxia and inflammatory stimuli [20]. Distinct from the other three isoenzymes localized in cytoplasm, PFKFB3 is mainly localized in the nucleus of cancer cells. Nuclear targeting PFKFB3 not only increases cell proliferation [23], our former study also showed it played a promotional role in regulating autophagy in renal cancer cells [22], and PFKFB3 deprivation reduced autophagy in rhabdomyosarcoma cells and malignant pleural mesothelioma cells [24,25]. PFK-15, an inhibitor of PFKFB3, promotes lipophagy and chemosensitivity in gynecologic cancers [26]. Furthermore, the inhibition of PFKFB3 has been reported to reduce pathological angiogenesis [27], improve chemotherapy [28], and conquer sorafenib resistance [29]. Therefore, it is possible to utilize PFKFB3 as a target for cancer therapy and overcome chemotherapeutic drug resistance.

Oxaliplatin (Oxa), a third-generation platinum analog used as standard therapeutic agent for both resected and metastatic CRC, has been reported to effectively regulate cell proliferation, apoptosis, and autophagy. However, the growing resistance and unneglectable side-effects are major risks of Oxa in CRC patients, which make it urgent to discover new therapy targets or strategies. Glycolysis has been shown to be associated with drug resistance [30], and another study showed that the knockdown of PKM2 (pyruvate kinase M2 type; another rate-limiting glycolytic enzyme) notably reversed the resistance of Oxa [31]. However, whether PFKFB3 plays a role in regulating Oxa-induced autophagy, and the effect of PFKFB3 inhibition on Oxa’s cytotoxicity have not been investigated. In the present study, we found Oxa activated autophagy concomitant with the up-regulation of PFKFB3 expression. Furthermore, the inhibition of PFKFB3 blocked the Oxa-induced autophagy, and enhanced the cytotoxicity of Oxa.

## 2. Results

### 2.1. Oxa Shows Effectively Cytotoxic Effect in SW480 Cells

**Figure 1:**
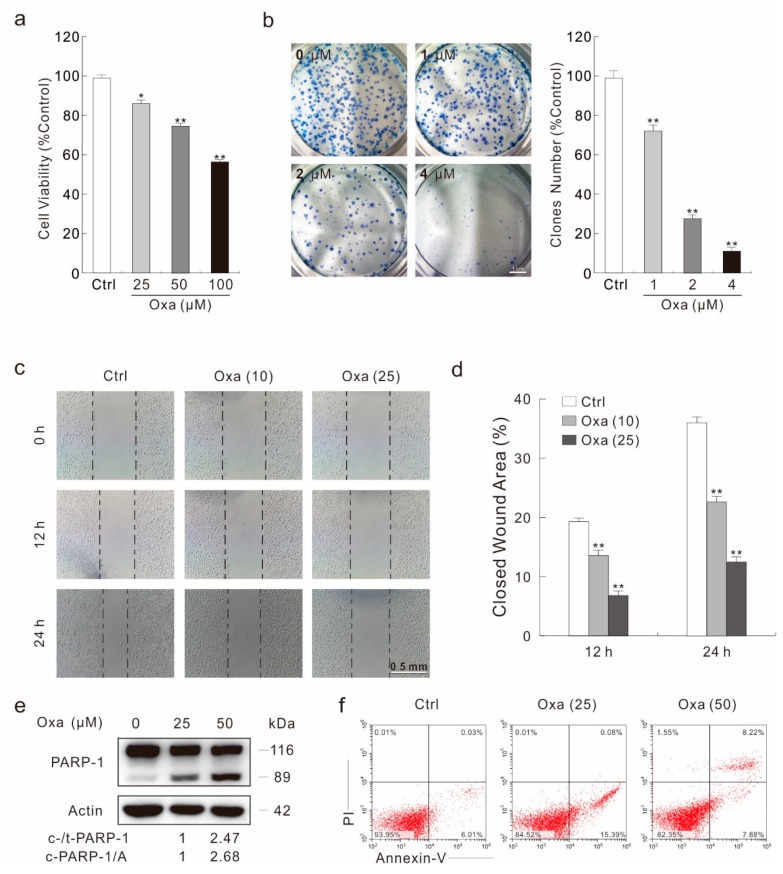
Oxa shows obvious cytotoxicity in SW480 cells. (**a**) SW480 cells were treated with Oxa for 24 h; cell viability was analyzed by a MTS assay as described in Materials and Methods. (**b**) A colony growth assay was performed with different doses of Oxa. (**c**,**d**) A wound healing assay was carried out with Oxa, and images were taken at indicated time points (100 magnification). The wound area was analyzed using Photoshop software and the relatively closed area of the wound was shown in the histogram (**d**). (**e**) Cell lysates were prepared and analyzed by immunoblotting after treating SW480 cells with Oxa for 24 h. (**f**) Following treatment of the cells with Oxa for 24 h, the apoptosis was determined by flow cytometry (AV-positive). For histogram results, data were presented as mean ± S.D. and were representatives of three independent experiments (* *p* < 0.05 vs. control, and ** *p* < 0.01 vs. control). Similar experiments were repeated three times.

As Oxa is a widely used anti-tumor drug, its cytotoxic effect has been monitored by a variety of methods. In a MTS assay, Oxa obviously reduced the viability of SW480 cells in a dose-dependent manner (Figure 1a). Meanwhile, it was shown to inhibit the cell proliferation by both a colony growth assay and a cell counting assay, respectively (Figure 1b; Figure S1A,B). Utilizing a wound healing assay, a simple but efficient method to detect the cell migration ability, we observed that cell migration was significantly decreased upon Oxa treatment (Figure 1c,d). Under an optical microscope, cells were well attached to the plate in the control group (Ctrl), whereas most of the Oxa-treated cells were rounded and loosely attached (Figure S1C), indicating that Oxa could induce cell death. PARP-1 is a cleavage target of caspase-3 in vivo, and cleavage of PARP-1 serves as a marker of cells undergoing caspase-dependent apoptosis [32]. Immunoblotting analysis showed that Oxa induced cleavage of PARP-1 (Figure 1e). Meanwhile, flow cytometry data confirmed that Oxa could induce apoptosis (Figure 1f). Furthermore, pretreatment of the pan-caspase inhibitor Z-V-FMK partly rescued the Oxa-induced cell viability loss (Figure S1D), suggesting that apoptotic cell death is not the only reason resulting in decreased cell viability during Oxa treatment.

### 2.2. Oxa Enhances Autophagic Flux

**Figure 2:**
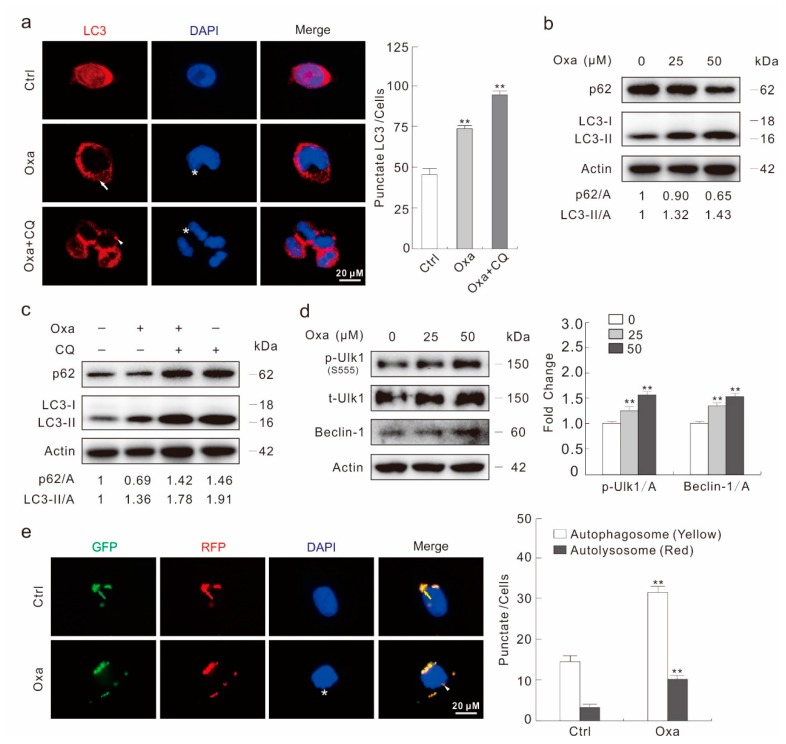
Oxa induces autophagy in SW480 cells. (**a**) Immunofluorescence using the antibody of LC3 was performed in SW480 cells following treatment with Oxa (25 μM hereafter, or otherwise indicated) in the presence or absence of CQ (20 μM hereafter) for 2 h (1000 magnification). The numbers of the punctate LC3 in each cell were counted, and at least 30 cells were included for each group. (**b**–**d**) Cells were treated with indicated dose of Oxa for 2 h in the presence or absence of CQ. Cell lysates were subjected to immunoblotting with the antibodies indicated. (**e**) After transfection with GFP-RFP-LC3 plasmids for 24 h and split onto coverslips then cultured overnight, SW480 cells were treated with or without Oxa for 2 h (1000 magnification). The number of the yellow and red dots in each cell was counted, and at least 20 cells were included for each group. Data represent three independent experiments. ** *p* < 0.01 vs. control.

Utilizing fluorescence microscopy, an accumulation of LC3 punctate staining was observed in Oxa-treated cells (Figure 2a). The plasmids expression green fluorescent protein (GFP) and LC3 fusion protein were transfected to SW480 cells and then treated with Oxa for 2 h. Similarly to the LC3 staining results, Oxa obviously increased the punctate staining of GFP-LC3 (Figure S2A,B). Furthermore, the addition of autophagic flux inhibitor chloroquine (CQ) increased both punctate staining of LC3 and GFP-LC3 (Figure 2a; Figure S2A,B). The immunoblotting analysis revealed that the Oxa treatment increased the ratio of LC3-II to Actin relative to control cells in a concentration-dependent manner (Figure 2b). Moreover, Oxa decreased the protein level of p62/SQSTM1, a selective substrate of autophagy (Figure 2b). To further analysis whether Oxa could induce the autophagic flux, CQ was utilized in the immunoblotting analysis. As expected, the addition of CQ further increased the LC3-II level and blocked the degradation of p62 (Figure 2c), suggesting the autophagic flux was enhanced under Oxa treatment. As Oxa showed no obvious affection of either LC3 and p62 expression when monitored by real-time PCR (Figure S2C), the protein level change of LC3 and p62 is a post-transcriptional event. In addition to the up-regulation of both Beclin-1 (another protein critical to autophagy process) and phosphorylated-Ulk1 (S555, p-Ulk1) (Figure 2d), Oxa also down-regulated the mTOR phosphorylation level and increased AMPK pathway activity, respectively (Figure S2D). Besides, we also carried out the GFP-RFP-LC3 assay, which is based on the different pH stability between GFP and RFP fluorescent proteins [10]. Oxa treatment not only increased the autophagosome dots (yellow), but also the autolysosome dots (red), indicating that Oxa aroused complete autophagic flux (Figure 2e). The aforementioned data indicated that Oxa could be regarded as an inducer of autophagy in SW480 cells.

Two widely used inhibitors of autophagy, 3-Methyladenine (3-MA) [33] and CQ, partly rescued the cell viability loss aroused by Oxa at the 24 h time point (Figure S3A). Meanwhile, both of them suppressed Oxa-induced PARP-1 cleavage, indicating that autophagy inhibitors rescued the apoptotic cell death aroused by Oxa (Figure S3B). To confirm these results, we knocked down two important autophagy genes, Ulk1 and LC3, respectively. Though deprivation of Ulk1 or LC3 failed to increase the cell viability, the elimination of the Ulk1 and LC3 expression partially protected Oxa-induced cell viability loss (Figure S3C). Meanwhile, the deprivation of either gene inhibited the Oxa-induced PARP-1 cleavage (Figure S3D). These findings indicated that autophagy was involved in Oxa-induced caspase-dependent apoptosis.

### 2.3. Oxa Enhances the Expression of PFKFB3

**Figure 3:**
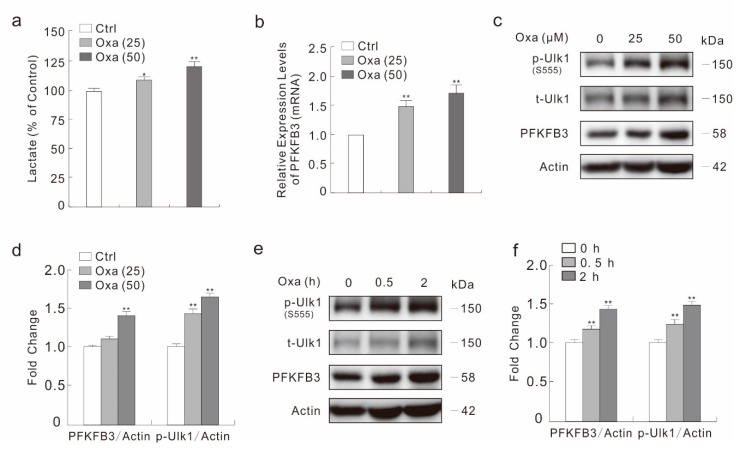
Oxa up-regulates the expression of PFKFB3. (**a**) Following treatment with Oxa for 6 h, culture media were collected and subjected to lactate assay. (**b**) Real-time PCR was performed to detect the mRNA expression of PFKFB3 following treatment with Oxa for 6 h. (**c**–**f**) SW480 cells were treated with Oxa (**e**: 50 μM) upon to 2 h (**c**: 2 h). Cell lysates were subjected to immunoblotting with the indicated antibodies, and the relatively ratios of PFKFB3 and p-Ulk1 to Actin were shown in the histogram graphs. * *p* < 0.05 vs. control, and ** *p* < 0.01 vs. control. Data represent three independent experiments.

Our former studies indicate that PFKFB3 participate in regulating autophagy and apoptosis [22,34], and other groups report that glycolytic enzymes are associate with drug resistance [30,31]. Thus, we wondered whether PFKFB3 is involved in the cytotoxic effect of Oxa. We first used a lactate assay to monitor the effect of Oxa on secreted lactate, which is the final product of glycolysis. As shown in Figure 3a, Oxa increased the lactate level in a dose-dependent manner. Utilizing real-time PCR, we observed that Oxa up-regulated the mRNA level of PFKFB3 in response to Oxa stimulation (Figure 3b). Consistently, Oxa increased the expression of PFKFB3 at protein level in a dose-dependent manner at 2 h time point, and 50 μM Oxa up-regulated the PFKFB3 level by about 45% compared to control cells (Figure 3c,d). Furthermore, Oxa increased the protein level of PFKFB3 at all time-points detected, as well as the phosphorylation level of Ulk1 (Figure 3e,f).

### 2.4. Inhibition of PFKFB3 Suppresses Oxa-Induced Autophagy

**Figure 4:**
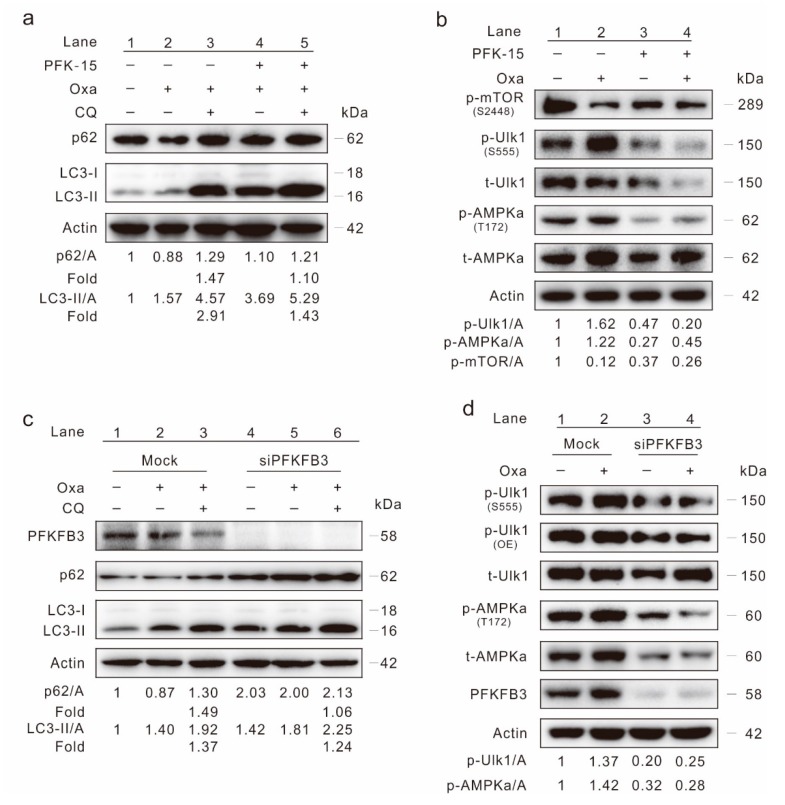
PFKFB3 inhibition lessens Oxa-induced autophagy. (**a**,**b**) Cell lysates were subjected to immunoblotting following treatment of SW480 cells with Oxa, or together with PFK-15 (6 μM; unless otherwise indicated) in the presence or absence of CQ for 2 h. (**c**,**d**) SW480 cells were transfected with the PFKFB3 siRNA or control siRNA (mock) for 48 h. Cell lysates were collected and subjected to immunoblotting following treatment with Oxa in the presence or absence of CQ for 2 h. All data were acquired from at least three independent experiments.

In our previous study, we have shown that the inhibition of PFKFB3 suppressed basal and rasfonin-induced autophagy in renal cancer carcinoma [22]. Here, we monitored the effect of PFKFB3 in regulating autophagy in SW480 cells. Utilizing an immunoblotting assay, PFK-15, a small molecule antagonist of PFKFB3, was found to increase LC3-II levels (Figure S4A). However, unlike general autophagy induction events, the p62 level did not decrease upon PFK-15 treatment (Figure S4A). Meanwhile, the addition of CQ failed to obvious accumulated LC3-II and block p62 degradation in the PFK-15-treated cells (Figure S4B). Although the LC3-II level in the Oxa/PFK-15 combination cells was higher than that in the Oxa-treated alone cells, the p62 level in the former group failed to be lower than the latter group (p62/A: lane 4 > lane 2), as well as the Ctrl cells (p62/A: lane 4 > lane 1) (Figure 4a). Though CQ increased the p62 and LC3-II levels in both Oxa- and Oxa/PFK-15-treated cells, the change fold (magnitude in changes for the levels of LC3-II/Actin and p62/Actin in the CQ-added cells compared to that of CQ-withdraw cells) of Oxa/PFK-15 was lower than Oxa cells after CQ treatment (Figure 4a; folds: lane 5 vs. 3). Furthermore, PFK-15 alone decreased the basal p-Ulk1 and p-AMPKα levels, and reduced the Oxa up-regulated p-Ulk1 and p-AMPKα levels as well (Figure 4b). Surprising, both Oxa and PFK-15 treated alone decreased the mTOR phosphorylation level (Figure 4b), thus the Akt-mTOR signaling pathway, the classically negative regulator of autophagy, may not be used to explain how PFK-15 inhibits basal and Oxa-induced autophagy.

To verify the results obtained from PFK-15 treatments, PFKFB3 in SW480 cells was deprived using target siRNA. PFKFB3 silencing obviously increased the p62 level, and the p62/Actin change fold in siPFKFB3 group was much lower than that in the mock group (Figure 4c; folds: lane 6 vs. 3; Figure S4C; folds: lane 4 vs. 2). Meanwhile, the change fold of LC3-II/Actin was also lower in the PFKFB3 silencing group than that in the mock control group (Figure 4c and Figure S4C). Similar to PFK-15 treatment, deprivation of PFKFB3 reduced both the basal and Oxa-increased p-Ulk1 as well as p-AMPKα levels (Figure 4d), indicating the AMPK pathway played a role in this process. Utilizing a lactate assay, both PFK-15 and silencing of PFKFB3 decreased the secreted lactate in SW480 cells (Figure S4D). The aforementioned data indicated that inhibition of PFKFB3 suppressed the autophagy induced by Oxa.

### 2.5. Inhibition of PFKFB3 Enhances the Cytotoxic Effect of Oxa

**Figure 5:**
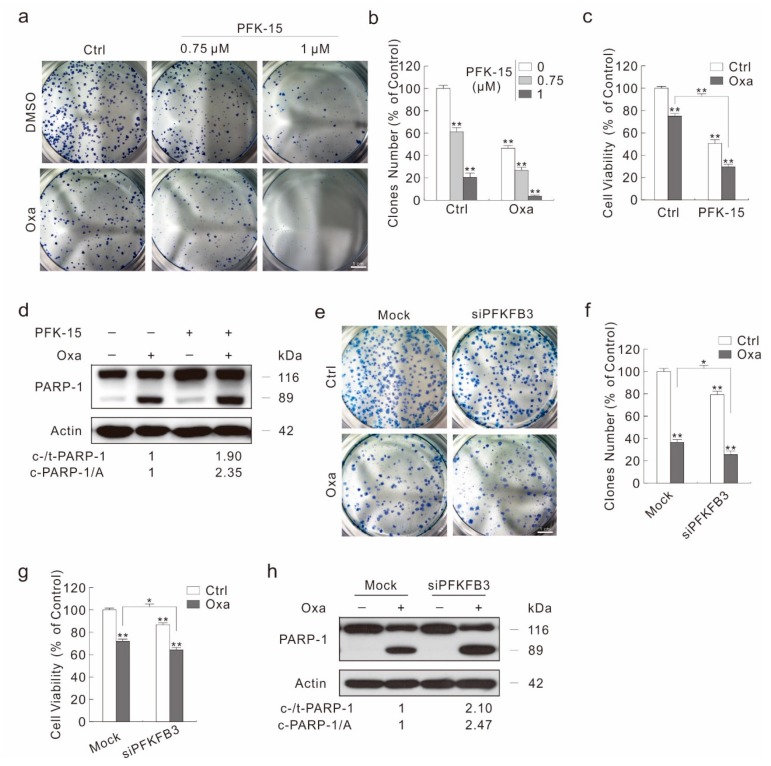
PFKFB3 inhibition enhances the cytotoxicity of Oxa. (**a**,**b**) Colony growth assay was performed with Oxa (1.5 μM) in the presence or absence of PFK-15. (**c**,**d**) Cell viability was analyzed by MTS assay following the treatment with Oxa in the presence or absence of PFK-15 for 24 h (**c**); cell lysates from cells treated same as (**c**) were subjected to immunoblotting with indicated antibodies (**d**). (**e**–**h**) SW480 cells were transfected with the PFKFB3 siRNA or control siRNA for 48 h. Colony growth assay was performed with Oxa (1.5 μM) (**e**), and relative clones number to control was shown in (**f**). Cell viability was analyzed by MTS assay following the treatment with Oxa for 24 h (**g**). Cell lysates were subjected to immunoblotting with indicated antibodies following treatment with Oxa for 24 h (**h**). For histogram graph data, * *p* < 0.05 vs. control, and ** *p* < 0.01 vs. control. Similar experiments were repeated three times.

As autophagy deprivation plays a protect role in the cytotoxicity of Oxa, and inhibition of PFKFB3 attenuates Oxa-induced autophagy, we then monitored the effect of PFKFB3 inhibition on the Oxa-induced cell viability loss and cell death. Unexpectedly, besides decreasing the cell proliferation in a dose-dependent manner when treated alone, PFK-15, even at low concentration, could further reduce the colony number decreased by Oxa (Figure 5a,b). Meanwhile, PFK-15 promoted the cell viability loss aroused by Oxa (Figure 5c). In addition, the combination of PFK-15 with Oxa further decreased the cell migration and cell proliferation compared to Oxa treated alone by wound healing assay and cell counting assay, respectively (Figure S5A–D). Although it promoted the Oxa-induced PARP-1 cleavage, a low dose of PFK-15 alone failed to cause obvious PARP-1 cleavage (Figure 5d), indicating that PFK-15 may induce other kinds of cell death to decrease the cell viability. In addition, a high dose of PFK-15 can induce apoptosis, and increased the Oxa-induced apoptotic cell death by flow cytometry assay (Figure S5E).

In order to avoid the off-target effect of PFK-15 resulted in these phenotypes, PFKFB3 was silenced using target siRNA. Similarly to the PFK-15 treatment, silencing of PFKFB3 not only reduced the basal, but also significantly enhanced the Oxa-decreased colony number (Figure 5e,f). Moreover, PFKFB3 silencing promoted Oxa-induced cell viability loss and apoptotic cell death (Figure 5g,h). Although PFKFB3 silencing alone decreased the cell viability, it failed to induce the obvious PARP-1 cleavage (Figure 5g,h). In addition, knock down of PFKFB3 significantly decreased the cell migration rate and cell proliferation, respectively (Figure S6A–D). In neither Ctrl nor Oxa-treated cells, transfection of mock control siRNA showed a significant difference compared to negative control cells (NC, none siRNA transfected) (Figure S6A,B). Thus, the transfection of PFKFB3 siRNA, not the transfection process itself, functioned the inhibitory effect. Taken together, inhibition of PFKFB3 promoted Oxa’s cytotoxic effect.

## 3. Discussion

A new finding in the present study is that Oxa induces autophagy with a concomitant promotion of PFKFB3 expression at both mRNA and protein levels in SW480 colon cancer cells. Target siRNA and pharmacologically deprivation of PFKFB3 blocks the ability of Oxa to induce autophagy. Moreover, inhibition of PFKFB3 promotes the cytotoxic effect of Oxa, such as cell viability, proliferation, and migration. Thus, PFKFB3 plays a protective role in SW480 cells upon Oxa treatment.

The Akt-mTOR and AMPK pathways are two mainly regulators in modulating autophagy intensity, by modifying the level of phosphorylated-Ulk1 (Serine 555; Ser555). Although the actually phosphorylation site is serine 556 (Ser556) according to the GenBank (AAC32326.1), Ser555 was widely used in many published research articles and antibody product names [17]. Thus, here we labeled it as phosphorylated-Ulk1 (Ser555) according to the tradition. In the present study, we observed that Oxa obviously increased the level of phosphorylated-Ulk1, and up-regulated the Beclin-1 level. Meanwhile, utilizing a series of methods, we demonstrated that Oxa functioned as an autophagy inducer in SW480 cells. Although Oxa might induce autophagy by inhibiting mTOR signaling and inducing AMPK signaling, the former pathway might not be possible to use as explanation of the autophagy regulated under the PFK-15 treatment. It is common sense that PFKFB3 inhibitor PFK-15 inhibits the mTOR activity, as PFKFB3 promotes cell proliferation and metabolism in a variety of cells [35,36]. However, despite the fact that the phosphorylated-mTOR levels were reduced, PFK-15 failed to induce autophagy in both basal and Oxa-treated cells. When it came to the AMPK signaling pathway, its activity was consistence with the autophagy intensity, as PFK-15 reduced the basal and Oxa-increased phosphorylated-AMPKα. Meanwhile, the changing pattern of phosphorylated-AMPKα, but not phosphorylated-mTOR, was similarly to the pattern of phosphorylated-Ulk1. Our former study also demonstrated that PFKFB3 regulated autophagy through the AMPK pathway [22]. In the future study, we will further investigate the role of the AMPK signaling pathway in regulating the PFK-15/Oxa modulated autophagy process.

Autophagy may play dual roles in regulating cell death, while autophagy promotes tumor cell survival to metabolic stress and radiotherapy/chemotherapy, prolonged/progressive autophagy can eventually lead to autophagic cell death [37,38]. The relationship between autophagy and apoptosis is complex, as autophagy can promote, suppress, or accompany apoptosis [39,40]. In addition, autophagy and apoptosis not only share some vital proteins, but also modulate the intensity of each other under certain circumstances [40,41]. It is interesting to notice that the inhibition of PFKFB3 displayed an opposite effect on Oxa-induced apoptosis compared to autophagy inhibition by either pharmacological or target siRNA. This may be due to, on the one hand, unlike the 3-MA/CQ treatment and knockdown of Ulk1/LC3, the inhibition of PFKFB3 affects autophagy through regulating the complex signaling network; on the other hand, PFKFB3 deprivation affects other cell biological processes, among which might contain apoptosis-contributing factors. For instance, the loss function of PFKFB3 shuts the glucose toward the pentose phosphate pathway (PPP), and renders cell apoptosis susceptible [34,36,42]. In future studies, we will monitor the effect of autophagy deprivation on Oxa’s cytotoxicity when treating in a prolonged manner, to further reveal the function of autophagy and autophagic cell death on chemotherapy.

It should not be ignored that PFKFB3 deprivation, especially treatment with PFK-15 under low concentrations, apparently reduces cell viability, but fails to induce caspase-dependent apoptosis. These phenomena are consistence with the former inference that the loss function of PFKFB3 renders cell apoptosis susceptible, but does not arouse apoptosis itself [42]. PFKFB3, which is highly expressed in cancer cells and rapid proliferating cells, has been reported to play essential roles in regulating metabolism, angiogenesis, as well as inflammation [35,43]. Consistently, we also displayed that the inhibition of PFKFB3 can effectively reduce cell proliferation and migration. Former studies indicated that PFKFB3 could localize in nucleus, and nuclear targeting of PFKFB3 promotes autophagy and proliferation [22,23]. Thus, whether the subcellular localization changing of PFKFB3 affects Oxa’s cytotoxicity will be continue to be explored.

In summary, the presented data clearly demonstrated that Oxa positively regulated autophagy and increased the expression of glycolytic enzyme PFKFB3. Inhibition of PFKFB3 attenuated Oxa-induced autophagy, and further enhanced its consequences on cell viability, proliferation, migration, as well as caspase-dependent apoptosis. Accordingly, the combination of low dose PFK-15 and Oxa could mimic the effect of high dose Oxa, thereby may reduce the side effects of Oxa and reduce the possibility of drug resistance. These results shed light on PFKFB3 and autophagy in affecting Oxa’s anti-tumor efficiency, and provide a theoretical basis regarding potential therapeutic targets and strategies for colon cancer.

## 4. Materials and Methods

### 4.1. Chemicals and Antibodies

Chloroquine diphosphate salt (CQ, C6628), and polyclonal antibodies against LC3 (L7543) were purchased form Sigma-Aldrich (St Louis, MO, USA). 3-methyladenine (3-MA, M129496) was acquired from Aladdin (Seattle, WA, USA). 1-(4-pyridinyl)-3-(2-quinolinyl)-2-propen-1-one (PFK-15, ab145859), and Oxaliplain (ab141054) were obtained from abcam (Cambridge, MA, USA). Z-VAD-FMK (FMK001) was purchased from R&D Systems (Minneapolis, MN, USA). The antibodies of phospho-Ulk1 (Ser555; 5869), total Ulk1 (8054), Beclin-1 (4122), PARP-1 (9542), phospho-AMPKα (Thr172, 2535), total AMPKα (2532), phospho-ACC (Ser79, 3661), phospho-mTOR (Ser2448, 2971), and total mTOR (2972) were obtained from Cell Signaling Technology (Beverley, MA, USA). Antibodies of p62 (sc-28359) were acquired from Santa Cruz Biotechnology (Dallas, TX, USA). The antibodies of PFKFB3 (13763-1-AP), PCNA (10205-2-AP), and β-Actin (60008-1-Ig) were purchased from Proteintech (Wuhan, Hubei, China). MTS (G1111) and PMS (P9625) were purchased from Promega Corporation (Madison, WI, USA) and Sigma-Aldrich, respectively.

### 4.2. Plasmids and siRNAs

The PFKFB3 plasmid was kindly provided by Dr. Yuqing Huo (Augusta University, GA, U.S.A.), and the GFP-LC3 and GFP-RFP-LC3 plasmids are the kind gifts of Dr. Tamotsu Yoshimori (Osaka University, Japan). The siRNA specific for human MAP LC3β (sc-43390), PFKFB3 (sc-44011), and Ulk1 (sc-44182) were purchased from Santa Cruz Biotechnology along with the control siRNA (sc-37007).

### 4.3. Cell Culture, Plasmid Transfection and siRNA Interference

SW480 cells obtaind from ATCC (Manassas, VA, USA) were grown in DMEM medium containing 10% fetal bovine serum (GIBCO, Grand Island, NY, USA), and 1% antibiotics. For plasmid transfection, cells of 60% confluence were transfected using Attractene (QIAGEN, Hilden, Germany) according to the manufacturer’s protocol. Cells were split and cultured overnight before subjecting to different treatments after transfection for 36 h. For siRNA interference, cells of 30% confluence in the medium without antibiotics were transfected using DharmaFECT (Dharmacon, T2001, Denver, CO, USA) following the manufacturer’s instructions. After culture for 48 h, cells were split and cultured overnight before exposure to indicated treatment.

### 4.4. Immunoblotting Analysis

After spit and cultured overnight to reach 70% to 80% confluence, cells were exposed to indicate treatments for appropriate period. Then the total protein homogenates were extract by Triton X-100/glycerol buffer [12]. After denaturation, the cell samples were separated on SDS-PAGE gels and then transferred to PVDF membrane. After blocking by 5% skimmed milk, immunoblotting was performed using appropriate primary antibodies and horseradish peroxidase-conjugated suitable secondary antibodies. The blots were detected with enhanced chemiluminescence (Pierce Chemical, 34080, Rockford, IL, USA).

### 4.5. Cell Viability Assay (MTS)

Cells were plated in 96-well plates (7500 cells per well) in 100 µL complete culture medium. After overnight culture, the medium was replaced with phenolic red-free complete medium contained indicated chemicals. After cultured for indicated period, 20 μL MTS/PMS (20:1) was added to each well, and the cell viability was determined by the amount of 490 nm absorbance using the microplate reader.

### 4.6. Colony Growth Assay

Cells were seeded at 300 cells/mL concentration in 6-well plate and cultured with complete medium for two weeks to allow colony growth under appropriate treatment. After 4% paraformaldehyde fixation and trypan blue (contain 1% Trion X-100) stain, pictures were taken by camera and then the numbers of colony were calculated by Image J software.

### 4.7. Wound Healing Assay

After plated and cultured overnight to reach 70% to 80% confluency, cells were scraped with a 20 μL-pipette tip. Culture medium was discarded and cells were washed with 37 °C warmed PBS for twice to remove the damaged and detached cells. After addition of fresh medium (with 5% FBS and 1% antibiotics) and indicated treatment, the wound closure was monitored by microscopy at different time points. The migration was determined using the Photoshop software as an average closed area of the wound relative to the initial wound area after wounding.

### 4.8. Lactate Assay

Equal number of cells were plated and cultured in 6-well plates overnight, and then the suspension was collected and subjected to lactate assay after indicated treatments. Levels of the secreted lactate were determined using the lactate assay kit (Megazyme, K-DATE, Co. Wicklow, Ireland) following the manufacturer’s protocol. Then the relative lactate levels were determined by dividing the Actin protein level harvested from the cells of the same pore as the suspension came from.

### 4.9. Fluorescence Microscopy

Cells were plated on glass cover slips and cultured overnight, and then performed the indicated treatments. Cells on glass cover slips were washed with Ca^2+^- and Mg^2+^-free PBS (CMF-PBS), fixed with freshly prepared 4% paraformaldehyde for 12 min. For GFP-LC3 and GFP-RFP-LC3 transfection cells, cells were then immersed in VECTASHIELD with DAPI (VECTOR, H1200, Burlingame, CA, USA) after washing three times. Otherwise, cells were permeabilized incubation with CMF-PBS containing 0.1% Triton X-100 and 0.5% BSA for 5 min. After being washed three times with CMF-PBS, cells were incubated with the indicated primary antibodies (diluted with CMF-PBS containing 0.1% Triton X-100 and 0.5% BSA) and then with appropriated secondary antibodies (diluted in CMF-PBS containing 0.5% BSA). Cells were then immersed in VECTASHIELD with DAPI after washing three times. Images were acquired via fluorescence microscopy (Nicon, Melville, NY, USA). The number of the punctate LC3 and indicated fluorescence dots were counted manually.

### 4.10. Flow Cytometry Assay

SW480 cells were treated with the indicated compounds, then trypsinized and harvested (keeping all floating cells), washed with PBS buffer, followed by incubation with fluorescein isothiocyanate-labeled annexin V (FITC) and propidium iodide (PI) according to the instructions of an Annexin-V-FITC Apoptosis Detection Kit (Biovision Inc., Milpitas, CA, USA, K101-100) and analyzed by flow cytometry (FACSAria, Becton Dickinson, Franklin Lakes, NJ, USA).

### 4.11. RNA Extraction and Real-Time PCR Analysis

The total cellular RNA was extracted using TRIzol reagent (Invitrogen, 15596-018, Waltham, MA, USA), and then reversely transcribed using PrimeScriptTM RT reagent Kit (TaKaRa, DRR037A, Dalian, Liaoning, China) according to the manufacturer’s protocol. Primer sequences used for amplification were as follows (Table 1):

Real-time PCR (CFX96^TM^; Bio-Rad, Hercules, CA, USA) was initiated with a 10 min denaturation at 95 °C in a final volume of 20 μL. The cycle profile was 95 °C (15 s), 60 °C (45 s), and 72 °C (1 min) for up to 40 cycles. Three repetitions were set for each sample, and the data were calculated by means of 2^−∆∆Ct^ based on the internal control of β-Actin.

### 4.12. Statistical Analysis

The normally distributed data were shown as mean ± S.D. (standard deviation). Student’s *t*-test was used to evaluate the statistically significant differences between two groups. Multigroup comparisons of the means were conducted by one-way analysis of variance with a post hoc Student–Newman–Keuls test. For the non-normally distributed data in the electron microscopy, results were show as mean, and analyzed using the Friedman test. Values of *p* < 0.05 were considered as statistically significantly different.

## Figures and Tables

**Table 1:** Primer sequences for Real-time PCR analysis.

Gene	Forward/Reverse	Nucleotides
PFKFB3	Forward (5′→3′)	GTGCCTTAGCTGCCTTGAGA
	Reverse (5′→3′)	CCGACTCGATGAAAAACGCC
LC3	Forward (5′→3′)	TAGAAGGCGCTTACAGCTCAAT
	Reverse (5′→3′)	ACTGACAATTTCATCCCGAACG
p62	Forward (5′→3′)	CATCGGAGGATCCGAGTGTG
	Reverse (5′→3′)	TTCTTTTCCCTCCGTGCTCC
β-Actin	Forward (5′→3′)	GCCTGACGGCCAGGTCATCAC
	Reverse (5′→3′)	CGGATGTCCACGTCACACTTC

## Supplementary Materials

Supplementary materials can be found at https://www.mdpi.com/1422-0067/20/21/5415/s1. Figure S1. Oxa inhibits cell proliferation and induces apoptosis. (A,B) Equal amount SW480 cells were planted and cultured overnight, and then pictures were taken at indicated time points with or without Oxa (10 μM) treatment (100 magnification). The cell numbers were shown in the histogram (B). (C) SW480 cells were treated with Oxa (10 μM), and then the morphology of cells were observed by optical microscopy (400 magnification). (D) Cell viability was analyzed by MTS assay with Oxa for 24 h in the presence or absence of Z-V-FMK (20 μM). For histogram results, data were presented as mean ± S.D. and all results were representatives of three independent experiments. NS indicated of none significant, * *p* < 0.05 vs. control, ** *p* < 0.01 vs. control. Similar experiments were repeated three times. Figure S2. Oxa stimulates autophagy. (A,B) After transfection with GFP-LC3 plasmid for 24 h, cells were split onto coverslips and cultured overnight. Following treatment with Oxa (25 μM; unless otherwise indicated) in the presence or absence of CQ (20 μM; unless otherwise indicated), cells were fixed by 4% paraformaldehyde for 12 min at room temperature (RT), and then visualized by fluorescence microscopy (1000 magnification). The numbers of the punctate GFP-LC3 in each cell were counted, and at least 30 cells were included for each group. (C) Real-time PCR was performed to detect the mRNA expression of LC3 and p62 following treatment with Oxa for 6 h. (D) After treating with or without Oxa (50 μM) for 2 h, cell lysates were subjected to immunoblotting with the antibodies indicated. Data represent three independent experiments. NS indicated of none significant, ** *p* < 0.01 vs. control. Figure S3. Inhibition of autophagy attenuates Oxa-induced cell viability loss and apoptosis. (A) SW480 cells were treated with indicated compounds (3-MA, 2 mM; CQ, 20 μM) for 24 h, cell viability was analyzed by MTS assay. (B) Cell lysates were prepared after indicated treatment for 24 h, and then subjected to immunoblotting with the antibodies indicated. (C,D) Following transfection with Ulk1 or LC3 targeted siRNAs for 48 h, SW480 cells were treated with Oxa for 24 h. Cell viability was analyzed by MTS assay (C), and cell lysates were prepared to immunoblotting with the antibodies indicated (D). Similar experiments repeated three times. * *p* < 0.05 vs. control, and ** *p* < 0.01 vs. control. Figure S4. Inhibition of PFKFB3 attenuates autophagy. (A,B) SW480 cells were treated with indicated dose of PFK-15 (B: 6 μM) in the presence or absence of CQ for 2 h. (C) Following transfection with PFKFB3 targeted siRNAs for 48 h, SW480 cells were treated with or without CQ for 2 h. Cell lysates were prepared and subjected to immunoblotting with the antibodies indicated. (D) Cell culture media from (A: PFK-15, 6 μM) and (C) were collected and performed lactate assay. Data represent three independent experiments. Figure S5. PFKFB3 inhibition depresses proliferation and migration. (A,B) Wound healing assay was carried out in PFK-15-treated (3 μM) SW480 cells with 10 μM Oxa, and images were taken at 0 h, and 24 h (100 magnification). The wound area was analyzed using Photoshop software and the relatively closed area of the wound was shown in the histogram. (C,D) Equal amounts of SW480 cells were planted and cultured overnight, and then pictures were taken at indicated time points with PFK-15 (3 μM) in the presence or absence of Oxa (10 μM; 100 magnification). (E) Following treatment of the cells with Oxa in the presence or absence of PFK-15 (12 μM) for 24 h, the apoptosis were determined by flow cytometry (AV-positive). * *p* < 0.05 vs. control, and ** *p* < 0.01 vs. control. Data represent three independent experiments. Figure S6. Silencing of PFKFB3 depresses proliferation and migration. SW480 cells were transfected with either siCtrl (mock), siPFKFB3 siRNAs or none siRNA (NC) for 48 h. (A,B) Wound healing assay was carried out with 10 μM Oxa, and the relatively closed wound area was shown in the histogram (100 magnification). (C,D) Equal amounts of cells were planted and cultured overnight, and then pictures were taken at 48 h time point with or without Oxa (10 μM; 100 magnification), and the cell number was shown in the histogram. NS indicated none significant, * *p* < 0.05 vs. control, and ** *p* < 0.01 vs. control. Data represent three independent experiments.
