# Supplementary figures and images for: PFKFB3 Inhibition Attenuates Oxaliplatin-Induced Autophagy and Enhances Its Cytotoxicity in Colon Cancer Cells
Source: Int J Mol Sci. 2019 Oct 30;20(21):5415. doi: 10.3390/ijms20215415 (PMC6862230; doi:10.3390/ijms20215415)

Figure S1

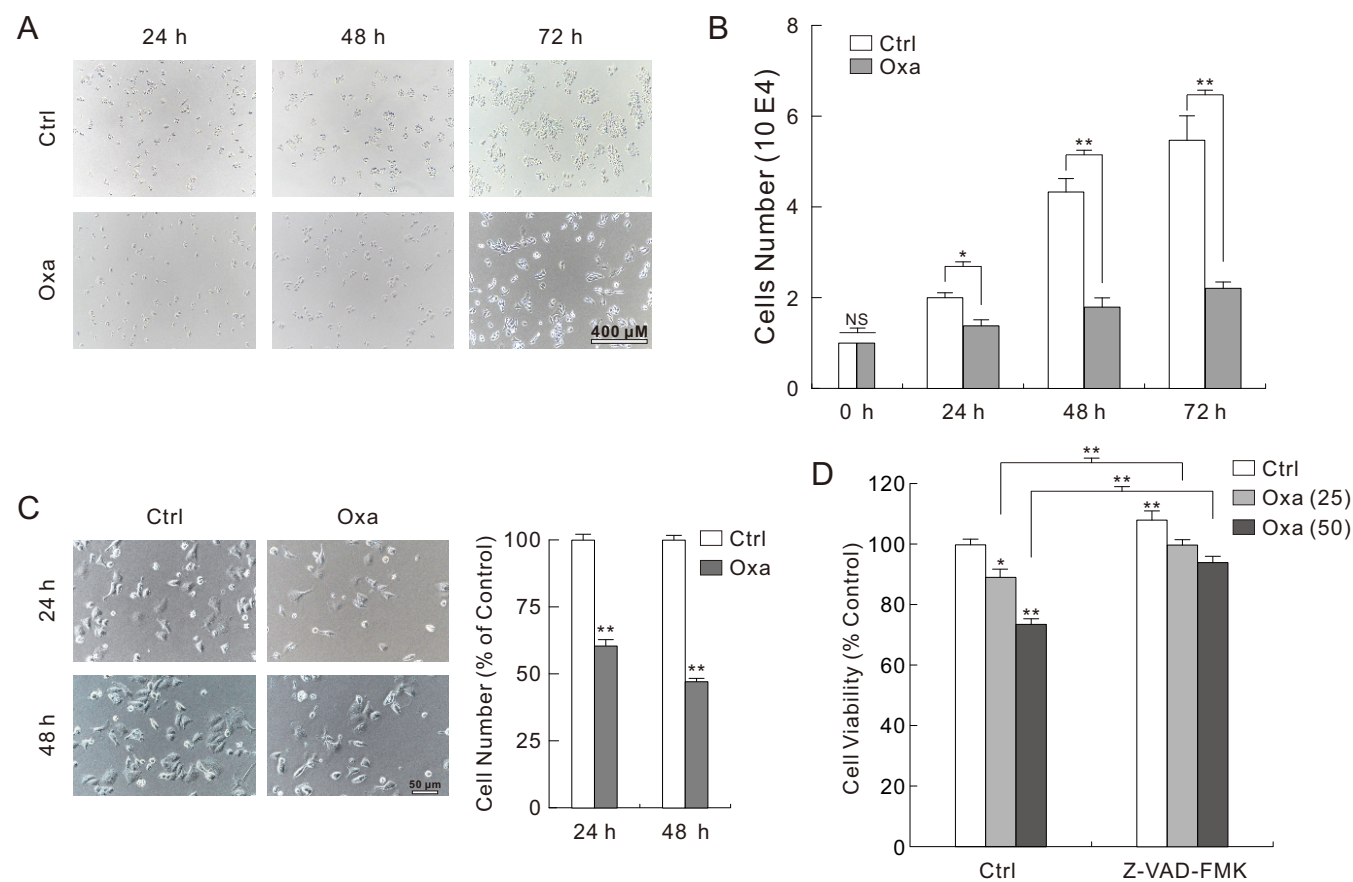

Supplement: Supplementary file 1 [file ijms-20-05415-s001.zip › Figure S1.pdf]

Figure S2

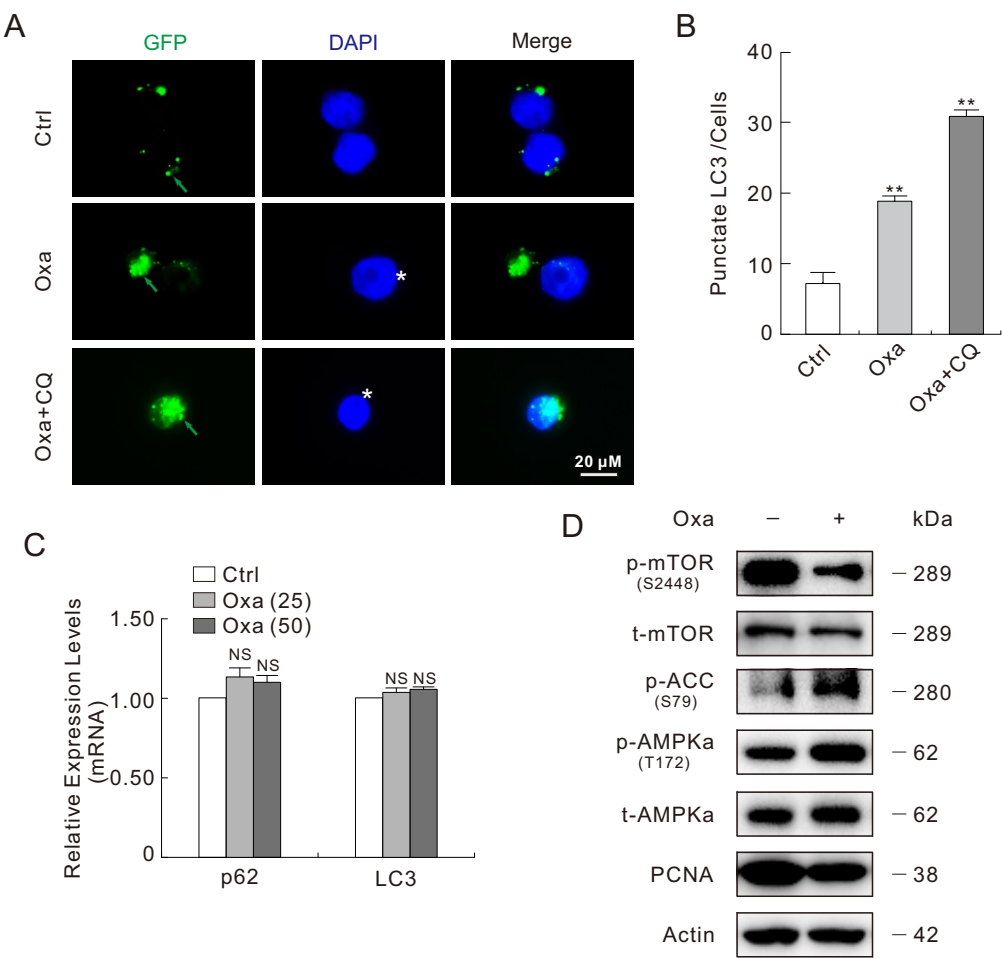

Supplement: Supplementary file 1 [file ijms-20-05415-s001.zip › Figure S2.pdf]

Figure S3

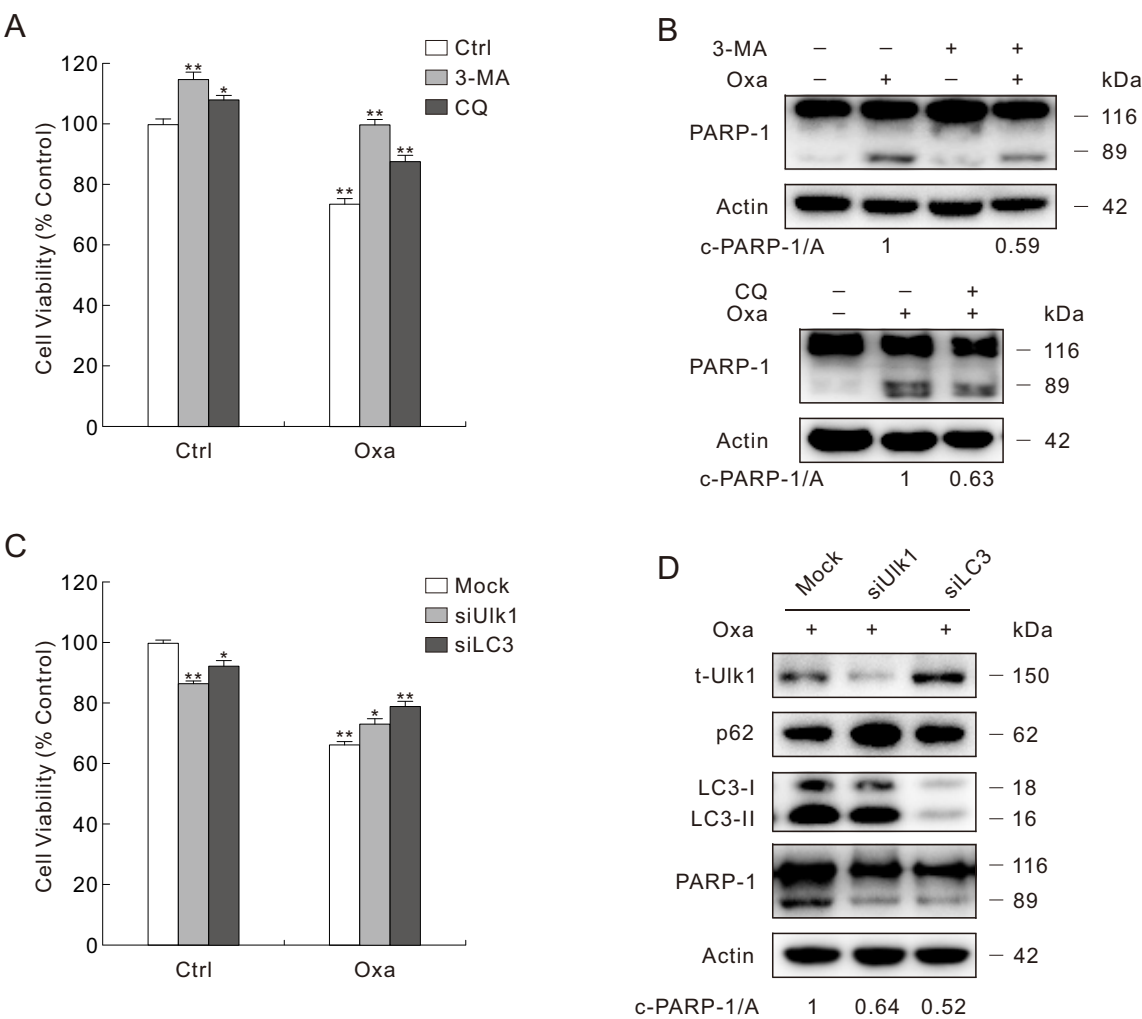

Supplement: Supplementary file 1 [file ijms-20-05415-s001.zip › Figure S3.pdf]

Figure S4

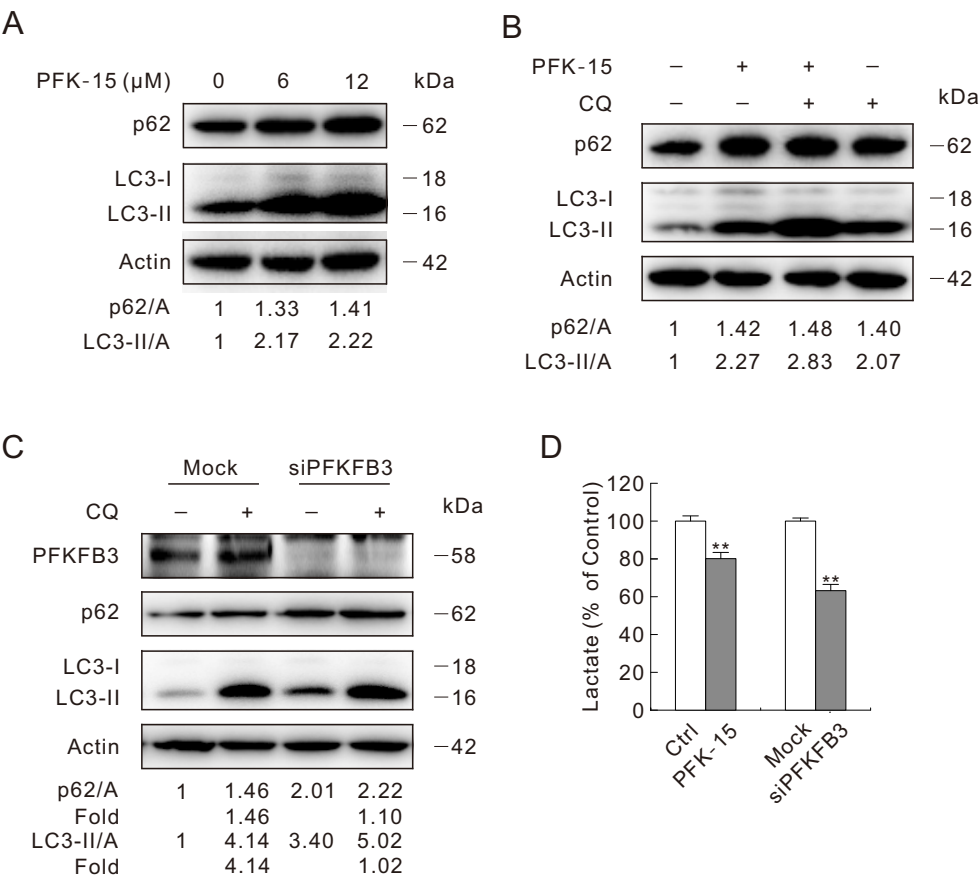

50  $\mu$ M 1h

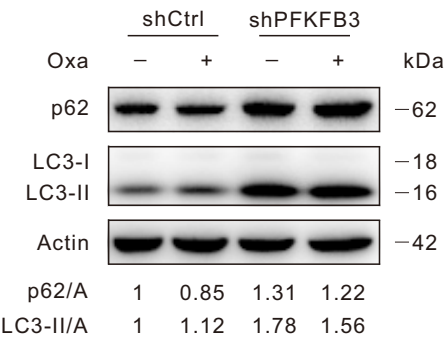

Supplement: Supplementary file 1 [file ijms-20-05415-s001.zip › Figure S4.pdf]

Figure S5

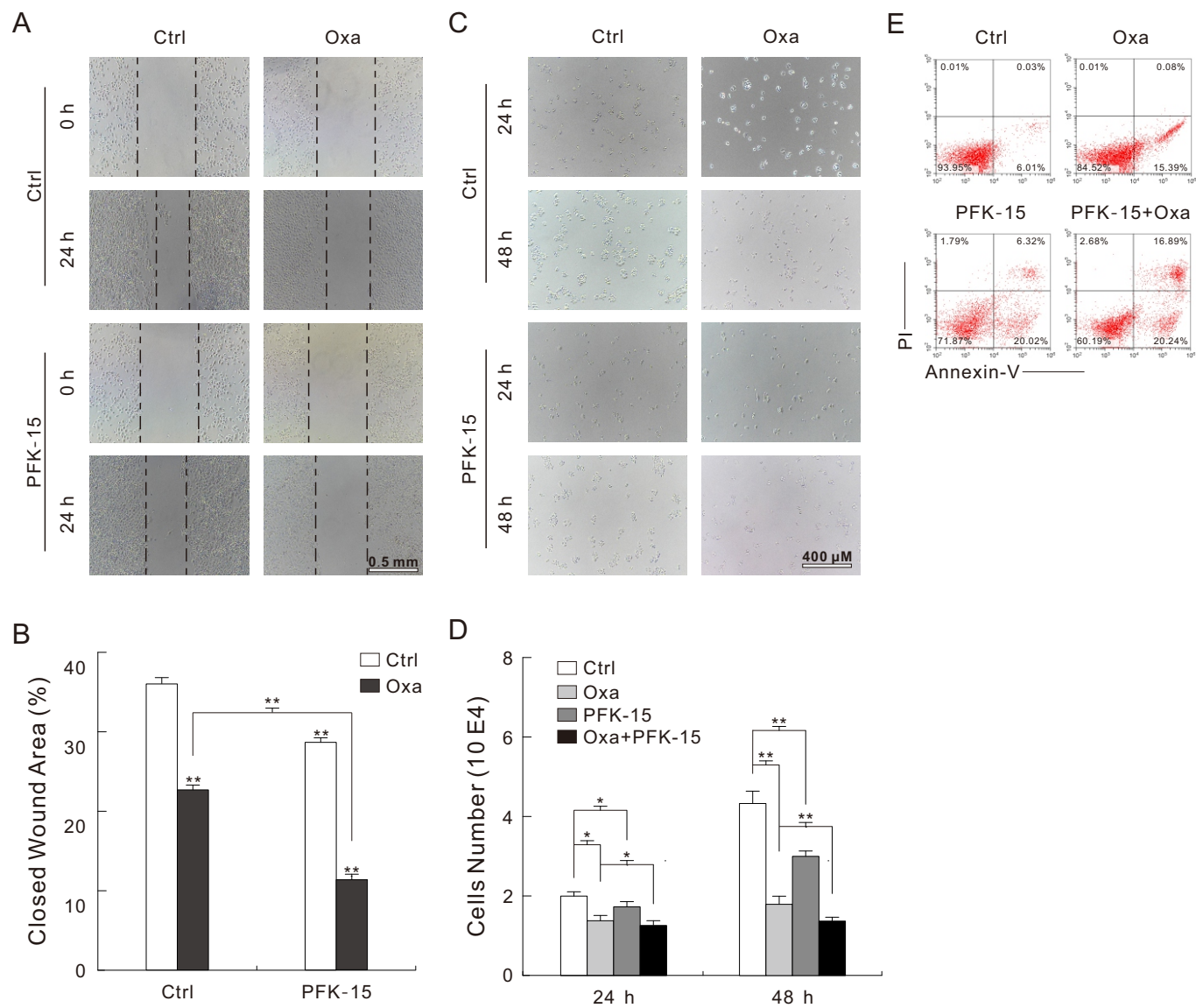

Supplement: Supplementary file 1 [file ijms-20-05415-s001.zip › Figure S5.pdf]

Figure S6

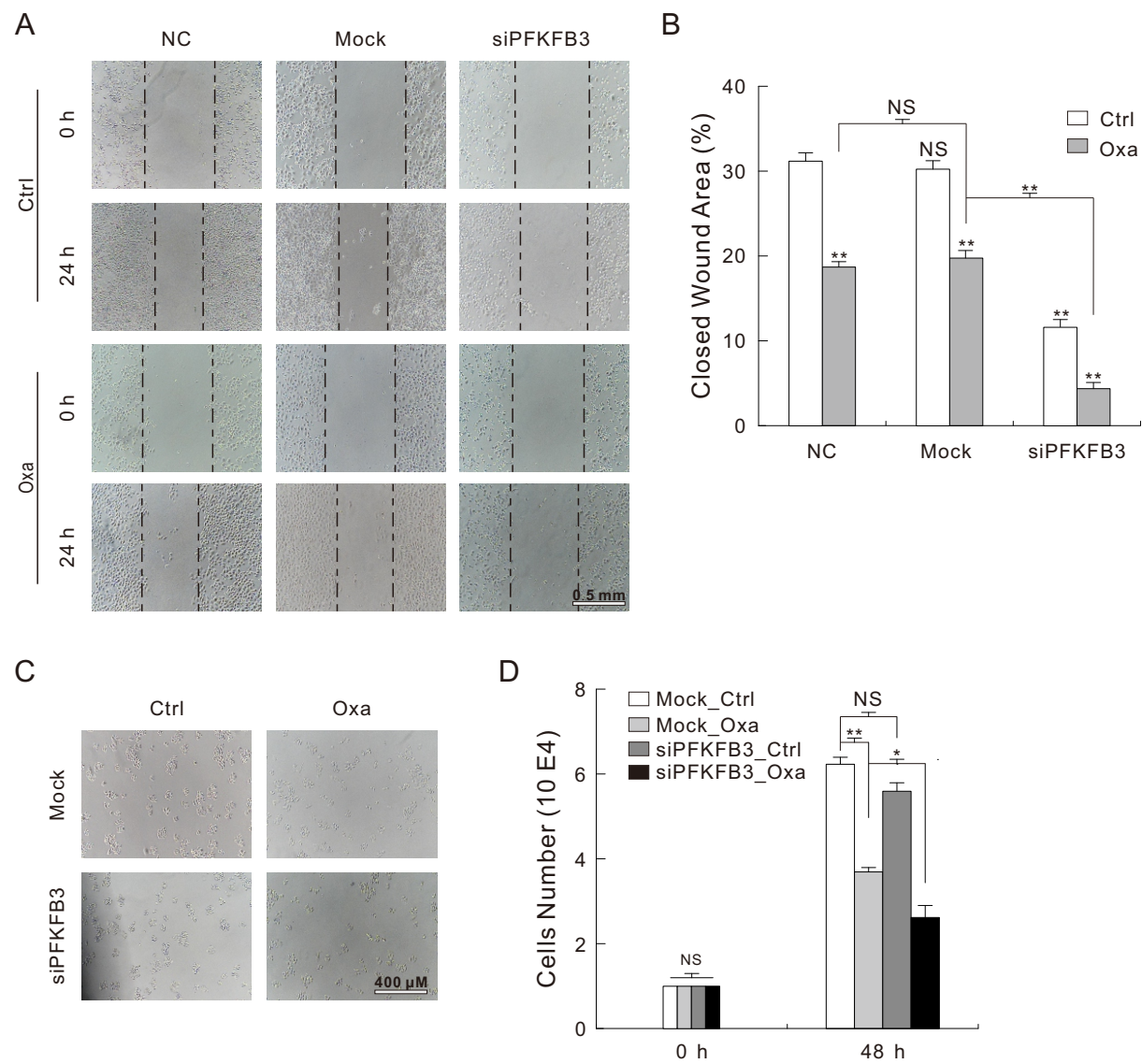

Supplement: Supplementary file 1 [file ijms-20-05415-s001.zip › Figure S6.pdf]
